# Effect of functional electrical stimulation combined with stationary cycling and sit to stand training on mobility and balance performance in a patient with traumatic brain injury: A case report

**DOI:** 10.1016/j.amsu.2021.103122

**Published:** 2021-12-02

**Authors:** Masoome Ebrahimzadeh, Noureddin Nakhostin Ansari, Scott Hasson, Ardalan Shariat, Seyed Ahmad Afzali

**Affiliations:** a Department of Physiotherapy, University of Social Welfare and Rehabilitation Sciences, Tehran, Iran; b Department of Physiotherapy, School of Rehabilitation, Tehran University of Medical Sciences, Tehran, Iran; c Research Center for War-affected People, Tehran University of Medical Sciences, Tehran, Iran; d Department of Physical Therapy, Augusta University, Augusta, GA, USA

**Keywords:** Functional electrical stimulation, Functional mobility, Balance performance, Traumatic brain injury, “Case report”

## Abstract

**Introduction and importance:**

This case study investigates the effects of functional electrical stimulation, stationary cycling, and sit-to-stand training in a patient with severe chronic traumatic brain injury.

**Case presentation:**

The participant was a 24-year-old man with a traumatic brain injury two years prior to the intervention described in this case report. The accident caused right hemiplegia, right foot drop, aphasia, and poor coordination of movement in both upper and lower limbs. He was using a wheeled walker for functional mobility and was receiving routine rehabilitation before the initiation of treatment. A four week intervention in this study included functional electrical stimulation of the quadriceps and tibialis anterior muscles combined with stationary cycling and sit-to-stand training.

**Clinical discussion:**

Active and passive range of motion of right ankle dorsiflexion, strength of ankle dorsiflexor, balance performance, and mobility were measured before and after the intervention. Active range of motion of right ankle dorsiflexion increased by 8°. In addition, manual muscle test and Brief-BESTest scores increased from 3+ to 5 and from 7 to 9, respectively. Walking speed over the 10-m distance and timed up and go test score improved.

**Conclusion:**

Functional electrical stimulation combined with stationary cycling and sit-to-stand training resulted in increased muscle strength and range of motion, improved balance performance, and improved mobility in an individual with a traumatic brain injury.

## Highlights

• Report of a unique multimodal rehabilitation technique in a patient with severe traumatic brain injury.
• Sit-to-stand is an appropriate exercise training for muscle strength and balance.
• Combination of functional electrical stimulation, stationary cycling, and sit-to-stand exercise should be considered for patients with traumatic brain injury.

## 1 Introduction

Traumatic brain injury (TBI) is one of the most important causes of death and chronic disability in the world and occurs primarily in adult men [1]. With improvements in medical science and technology, the mortality of these individuals has decreased. However, in severe cases of individuals who sustained a TBI and survived, extensive complications, such as cognitive and motor dysfunctions, psychiatric disorders, seizures, decline in quality of life, and increased economic cost were observed [2,3]. Various treatments have been suggested for reducing the disability and complications of TBI. The World Health Organization (WHO) emphasizes the active and dynamic process of rehabilitation for individuals with TBI [4]. Therefore, developing evidence-based treatment approaches and beneficial rehabilitation programs is necessary.

Functional electrical stimulation (FES) is utilized to improve muscle strength through the application of electric current through healthy peripheral motor nerves [5]. Studies have shown that FES training in the lower extremity can improve muscle strength, joint range of motion (ROM), and gait performance in patients who have had a stroke [6,7]. However, voluntary muscle contraction training (i.e., strength training) resulted in greater gains than FES training alone [8]. Cycling and sit-to-stand or stand-to-sit (STS) are motor tasks that are prerequisite exercises for mobility and walking [[9], [10], [11]]. Practicing both voluntary and involuntary muscle strengthening may improve balance control and the ability to walk independently, as these are the most important goals of rehabilitation for people with TBI and require intensive repetitive exercise. We assume that exercise augmented with FES can have beneficial outcomes. Therefore, the aim of this study was to investigate the effects of the simultaneous use of FES and cycling and STS exercise on mobility and balance performance in a patient with TBI.

## 2 Case presentation

### 2.1 Participant history

The participant in this study was a 24-year-old man who was in a motor vehicle accident that led to a severe TBI two years ago. According to the report of the spiral brain CT scan, the primary lesion was located in the left frontotemporal area due to contusion, and a few lacunar infarcts were seen in the left basal ganglia. Before the accident, he was an active member of a music band and was involved in bodybuilding activities. He was hospitalized for 48 days after the accident. Following discharge from the hospital, he received regular rehabilitation, including electrical stimulation of the wrist and knee extensors and ankle dorsiflexors, resistance training, and aerobic and endurance conditioning (e.g., walking on treadmill and stationary bike). At the time of the first visit to the research clinic, he could not independently walk or stand up from a chair and was using a wheeled walker for mobility and an ankle foot orthosis to prevent drop foot.

### 2.2 Clinical examination

The clinical examination was performed by an experienced physical therapist. The participant had right (RT) hemiplegia with full and strong grasping and gripping but without the ability to write. Other impairments were aphasia, bradykinesia, and dyscoordination of movements of RT upper (i.e., finger to nose) and lower (i.e., heel to shin) extremities. Also, deep tendon reflexes were increased with no spasticity in his muscles. He was dependent in some activities of daily living (ADL) (e.g., dressing, toilet use, and feeding).

## 3 Intervention

**Fig. 1:**
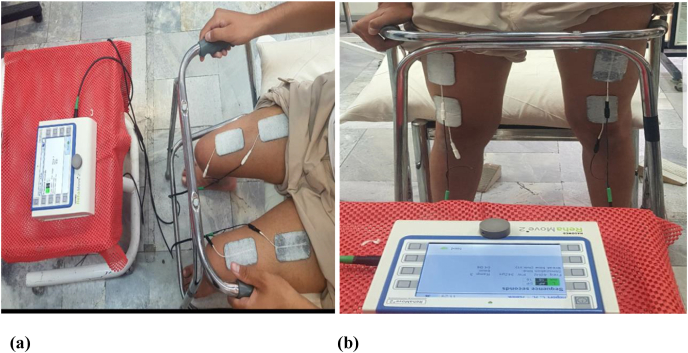
Standing up from a chair while functional electrical stimulation was applied on quadriceps of both legs.

**Fig. 2:**
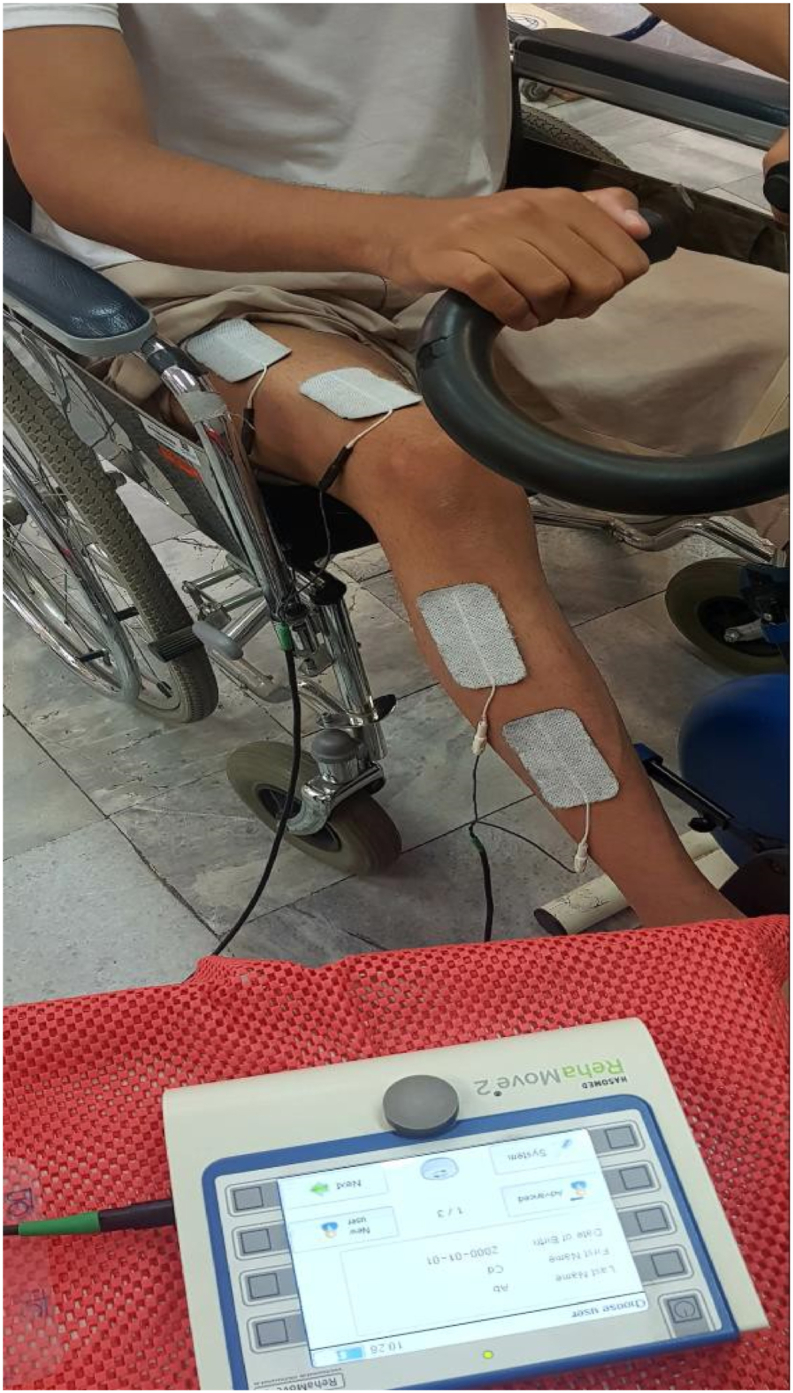
Cycling combined functional electrical stimulation Of right quadriceps and dorsiflexor muscles.

Intervention consisted of twelve sessions of stationary cycling combined with FES applied on the quadriceps (QC) and dorsiflexor muscles of the affected leg and STS exercise combined with FES applied on the QC muscles of both legs, three times a week (over a four-week period) (Fig. 1, Fig. 2). The electrical stimulation parameters were progressed by changing the pulse frequency from lower to higher (35–40 HZ), the duration of the wave from shorter to longer (300–450 μs), and the intensity (highest tolerable stimulation) [5,6]. Considering the patient's performance at baseline, three cycles of the stationary cycling program (15 min of actual cycling) and two cycles of the STS program (4 min of actual STS) were established in the first week of treatment. The details of the stationary cycling and STS programs are shown in Table 1; progression over four weeks is shown in Table 2. The stationary bike was set at 25 W for the entirety of the intervention. In the fourth and final week, the patient was able to do 30 min of cycling and 10 min of STS.

**Table 1:** Initial training parameters all performed with FES.

	**Warm up**	**Original**	**Cool down**	**Break**
SC X 3	1 min pedal	5 min pedal with FES	1 min pedal	2 min
STS X 2	30 sec STS	2 min STS with FES	30 sec STS	2 min

SC = stationary cycling; min = minutes; FES = functional electrical stimulation; STS = sit to stand/stand to sit; sec = second.

**Table 2:** Progression over the four-week intervention (SC and STS augmented with FES).

	First Week	Second Week	Third Week	Fourth week
SC	3 cycle (27 min)	4 cycle (36 min)	5 cycle (45 min)	6 cycle (52 min)
STS	2 cycle (10 min)	3 cycle (15 min)	4 cycle (20 min)	5 cycle (25 min)

SC = stationary cycling; min = minutes; FES = functional electrical stimulation; STS = sit-to-stand/stand-to-sit.

## 4 Outcome measures

Active and passive ankle ROMs were measured by goniometer, and the muscle strength of the ankle dorsiflexor in the affected leg was assessed by manual muscle testing (MMT). The Persian version of Brief-BESTest, which has a high inter-rater and intra-rater reliability in patients with stroke (ICC = 0.98 and ICC = 0.99 respectively) [12], was used to assess balance performance. In addition, walking speed over a 10-m distance (WS10 M) was measured prior to (at baseline) and after the intervention. The results of a previous study indicated a very high inter-rater reliability (ICC = 0.999) and an excellent concurrent validity for the WS10 M in patients with TBI [13]. Mobility activity was also evaluated by the timed up and go (TUG) test, which has excellent test-retest reliability in patients with stroke (ICC >0.95) [14]. The participant used his walker to perform the WS10 M and TUG tests. Measures were assessed at baseline and at the end of the sixth and twelfth sessions of intervention. This case report is about the effects of a rehabilitation program and followed the CARE guideline [15,16].

## 5 Results

At the end of the sixth and twelfth sessions of post-treatment, passive ankle ROM had increased by 2° in comparison with baseline. Active ankle ROM had increased by 5 and 8° at the end of the sixth and twelfth sessions, respectively. The MMT of the affected ankle dorsiflexors improved from 3+ at baseline to 5 at the end of the sixth session. The WS10 M increased from 0.84 m/s at baseline to 0.92 m/s and 1.07 m/s in the sixth and twelfth sessions of treatment, respectively. The TUG time decreased from 46 seconds at baseline to 40 seconds in the sixth session and 28 seconds in the twelfth session. In addition, the Brief-BESTest score increased from 7 at baseline to 9 in the sixth session of treatment. However, it did not change from the sixth to the twelfth session. At the end of the intervention, the participant was able to walk 8 m and sit to stand and stand to sit independently. He was also able to dress by himself. At one-month follow-up, he was able to walk up and down stairs using a tripod cane. Details of the outcomes measures are presented in Table 3.

**Table 3:** Outcome measures.

	MMT	PROM	AROM	WS10MD	TUG	Brief-BESTest
Baseline	3+	10	4	0.84	46	7
Post-test (session 6)	5	12	9	0.92	40	9
Completion (session 12)	5	12	12	1.07	28	9

MMT = manual muscle test; PROM = passive range of motion; AROM = active range of motion; WS10 M = walk speed over 10 m distance; TUG = timed up and go test; Brief-BESTest = brief-balance evaluation system test.

## 6 Discussion

The purpose of this case report was to investigate the effects of a four week intervention combining FES with stationary cycling and STS training on balance performance and mobility in a patient with a severe TBI. The results revealed improvements in active ankle dorsiflexion and muscle strength (reducing foot drop), walking speed, and balance. There is conflicting evidence in the literature concerning the effects of lower limb FES on postural control and mobility. Lo et al. reported that cycling combined with FES decreased spasticity and improved postural control in individuals with stroke [11]. However, de Sousa et al. showed that FES with cycling did not improve mobility and muscle strength in individuals with stroke [17]. To the authors’ knowledge, this is the first time that STS has been used to improve strength and coordination between the trunk and lower limbs for the maintenance of center-of-mass stability [18]. STS competence has been shown to have a strong correlation with balance ability [19] and mobility in individuals with stroke [20]. Low-frequency and high-pulse amplitude electrical stimulation was previously used to increase contraction time and motor unit activation and to stimulate fatigue-resistance muscle fibers selectively [21,22]. It is possible that the simultaneous application of FES (involuntary strength training) combined with demanding exercise tasks (voluntary strength training) can improve the mechanical output of muscles. Also, the nociceptive cutaneous stimulation and sensory input produced by FES on one side and activation of the synergistic muscle pattern produced during stationary cycling and STS could modulate neural drive and central adaptation [23]. Therefore, the cumulative effects of FES combined with demanding volitional activity has the potential to improve muscle performance, walking ability, and balance control in this case.

## 7 Conclusion

This case study demonstrated that four weeks of FES combined with stationary cycling and STS has the potential to improve muscle strength and ROM, walking velocity, and balance performance in a patient with a severe TBI. Future studies with a large sample size and control group are recommended to gain a greater understanding of the effects of FES combined with tasks such as stationary cycling and STS.

## Ethical approval

The ethical committee approval was not needed as this is a case report type article. However, the written informed consent was obtained from the patient to publish the clinical data.

## Sources of funding

This study is not funded.

## Author contribution

ME: Study concept, design, data collection, writing the manuscript draft. NNA: Study concept, design, data validation, revising the manuscript, supervision. SH: Data validation, revising the manuscript. AS: Study concept, design, data collection, revising the manuscript. SAA: Data collection. All authors read and approved the final manuscript for submission.

## Declaration of competing interest

The authors report no conflict of interest.

## Registration of research studies

Not applicable.

## Guarantor

Masoome Ebrahimzadeh.

## Declaration of conflicting interests

The author(s) declared no potential conflicts of interests with respect to the research, authorship, and/or publication of this article.

## Patient’s consent

Written informed consent was obtained from the patient for publication of this case report and accompanying images. A copy of the written consent is available for review by the Editor-in-Chief of this journal on request.

## Research registration

Not applicable.

## Provenance and peer review

Not commissioned, externally peer-reviewed.
